# Amelioration of Astrocytic Dysfunction via AQP4/LRP1 Pathway by *Zizania latifolia* and Tricin in C6 Cells Exposed to Amyloid β and High-Dose Insulin and in Mice Treated with Scopolamine

**DOI:** 10.4014/jmb.2412.12026

**Published:** 2025-02-25

**Authors:** Seun-Ah Yang, Se-Ho Park, Eun-Hye Kim, Won-Bin Bae, Kwang-Hwan Jhee

**Affiliations:** 1 Department of Food Science and Technology, Keimyung University, Daegu 42601, Republic of Korea; 2 Department of Applied Chemistry, Kumoh National Institute of Technology, Gumi 39177, Republic of Korea

**Keywords:** C6 astroglial cell, amyloid β, GFAP, AQP4, LRP1

## Abstract

*Zizania latifolia* and its bioactive compound tricin have been recognized for their anti-inflammatory, anti-allergic, and anti-aging properties. However, the impact of *Z. latifolia* extract (ZLE) and tricin on astrocyte dysfunction, particularly related to disruptions in the amyloid β (Aβ) clearance pathway, has not been extensively studied. This research aims to explore the regulatory effects of ZLE and tricin on astroglial dysfunction, utilizing astrocytic differentiated C6 cells (passages 75~85) subjected to Aβ and high-dose insulin, as well as scopolamine-induced mice. Results revealed that ZLE (500 μg/ml) and tricin (1 μg/ml) significantly upregulated the expression of astrocyte proteins GFAP and AQP4, brain-derived neurotrophic factor (BDNF), low-density lipoprotein receptor-related protein 1 (LRP1), and matrix metalloproteinases (MMPs) in C6 cells treated with Aβ and high-dose insulin. Furthermore, oral administration of ZLE (100 and 300 mg/kg) and tricin (0.3 mg/kg) in mice led to an increase in acetylcholine (ACh) levels and upregulation of insulin-degrading enzyme (IDE), LRP1, and MMPs, while reducing the levels of acetylcholinesterase (AChE), Aβ and ApoE4. These findings suggest that ZLE and tricin may ameliorate Aβ and high-dose insulin-induced astrocyte dysfunction in C6 cells and scopolamine-treated mice, potentially through the AQP4/LRP1 pathway.

## Introduction

Astrocytes are the predominant glial cells within the central nervous system (CNS), playing a crucial role in maintaining brain homeostasis. They are integral to metabolic support, nutritional provision, ion and neuro-transmitter balance, blood-brain barrier regulation, and CNS defense mechanisms. Given these homeostatic responsibilities, astrocytes are intimately involved in the pathophysiology of neurological disorders. The balance between neuronal damage, neuroprotection, and regeneration critically influences the progression and outcomes of these diseases. Brain injuries, whether acute or chronic, elicit a specialized glial response known as reactive astrogliosis, characterized by significant morphological and functional changes in astrocytes.

Alzheimer’s disease (AD), an age-associated neurodegenerative condition, is defined by the accumulation of extracellular fibrillar amyloid β (Aβ) plaques and intracellular neurofibrillary tangles of hyperphosphorylated tau protein [1]. Pathological changes in astrocytes during Alzheimer’s disease (AD) highlight distinct temporal patterns. In the later stages of AD, astrogliosis becomes prominent, with reactive astrocytes closely associated with senile plaque formation. Supporting this, *in vitro* studies with cultured astrocytes have shown that exposure to Aβ1–42 oligomers activates astrocytes, resulting in increased expression of GFAP and IL-1β [2], emphasizing the critical role of astrocytes in modulating Aβ-induced neurotoxicity. However, recent studies using transgenic AD mouse models have revealed significant astrodegeneration occurring early in AD progression, even before plaque formation [3]. This early-stage astrodegeneration is often characterized by reduced GFAP expression, as observed in the hippocampus of AD animal models [3, 4]. Astrodegenerative changes, including decreased expression of astrocyte markers such as aquaporin-4 (AQP4) and connexins, have been reported in animal models of stress-induced depression [5, 6]. AQP4, a key astrocytic water channel in the brain, is crucial for memory function, and its deficiency is associated with increased Aβ plaque deposition and related memory deficits [7, 8]. Studies suggest that proper AQP4 function is essential for effective Aβ clearance, with its impairment or mislocalization disrupting glymphatic function and exacerbating Aβ accumulation [9, 10]. Additionally, low-density lipoprotein receptor-related protein 1 (LRP1) plays a vital role in Aβ clearance by mediating its uptake into astrocytes, facilitating its degradation within lysosomes [11, 12]. Astrocytes also secrete several proteases, such as neprilysin, endothelin-converting enzymes, and insulin-degrading enzyme (IDE), which further promote Aβ degradation and prevent its aggregation into plaques, a hallmark of AD [13, 14]. On the other hand, upregulated expression of apolipoprotein E4 (ApoE4), a significant genetic risk factor for AD, has been reported to accelerate dendritic spine loss and memory impairment, ultimately leading to cognitive decline in AD [15].

Recent studies have underscored the significant role of astrocytes and brain-derived neurotrophic factor (BDNF) in the development of Alzheimer's disease (AD). BDNF, a crucial neurotrophin, supports neuronal survival and function, and it is essential for synaptic plasticity, which is vital for learning and memory processes. In patients with AD, consistently lower levels of BDNF have been observed, correlating strongly with the severity of cognitive decline [16, 17]. Consequently, therapeutic strategies aimed at preserving or enhancing BDNF levels are being actively explored as potential interventions for AD.

In addition to BDNF, aquaporin-4 (AQP4) has emerged as a critical factor in maintaining normal astrocyte function, as evidenced by recent *in vivo* studies. However, research on AQP4 expression regulation in astroglial cells under Aβ-induced degenerative conditions is limited. Furthermore, the expression of astrocyte markers in these models has been inconsistent, influenced by variables such as cell passage number, differentiation state, and the specific characteristics of Aβ used, including concentration and solubility. The astroglial C6 cell line is frequently employed to study the effects of various compounds on AD-related pathology, including assessments of cell viability, metalloproteinase (MMP) expression, and BDNF levels [18-20]. Notably, Capoccia *et al*. [21] reported that inhibiting AQP4 in C6 cells impacts cell migration and apoptosis. In contrast, studies by Dolman *et al*. [22] and Yoneda *et al*. [23] found that AQP4 expression is absent in C6 cells. The C6 cell line, which resembles glial precursor cells, typically expresses low levels of GFAP, an astrocyte-specific marker [24]. Because C6 cells have the capacity to differentiate into astrocytes, astrocytic differentiation was required to express astrocyte marker [25, 26]. For instance, Goya *et al*. [26] demonstrated that treating C6 glioma cells with insulin promotes their differentiation into astrocytes by activating the enzyme glutamine synthetase, a key astrocyte marker. Building on these findings, insulin was utilized as a differentiation agent in our previous research to induce astrocytic properties in C6 glioma cells [27]. However, the use of insulin as a differentiation agent has its drawbacks. High concentrations of insulin have been shown to increase neuronal sensitivity to excitotoxicity. In rat neuron cultures, insulin exacerbated the toxic effects of excitatory amino acids, such as glutamate, underscoring the potential risks of using high doses [28, 29]. Additionally, elevated insulin levels have been linked to a significant reduction in AQP4 and GFAP expression in astrocytes. This downregulation impairs Aβ clearance, leading to its accumulation in the brain. The concurrent reduction in GFAP expression indicates a loss of astrocyte reactivity, which can further exacerbate neurodegenerative processes [30]. IDE plays a crucial role in regulating insulin levels by breaking it down after cellular internalization, thereby preventing its excessive accumulation. High insulin concentrations can compete with Aβ for degradation by IDE, impairing Aβ clearance [31]. This effect is further supported by findings from IDE knockout models, which show increased accumulation of Aβ peptides in the brain [32, 33]. Thus, elevated insulin levels not only reduce the expression of essential proteins like AQP4 and GFAP but also contribute to an environment that fosters the development of AD pathology. Consistent with prior research, our previous study demonstrated that low-concentration insulin treatment promoted cell differentiation and increased the protein expression of astrocyte markers, while high-concentration insulin treatment inhibited both the expression of these markers. Furthermore, when combined with amyloid beta, AQP4/LRP and AQP4/EAAC1 signaling pathways were significantly suppressed. Therefore, in this study, we established conditions to treat C6 cells with high-concentration insulin in conjunction with Aβ.

*Zizania latifolia* (Gramineae) and its primary bioactive compound tricin have demonstrated considerable potential across a range of pharmacological applications, including immunomodulatory [34], anti-allergy [35], anti-wrinkle [36], anticancer, and antidiabetic effects [37]. These diverse properties suggest that *Z. latifolia* and tricin hold promise as functional food ingredients for the prevention and management of various chronic conditions. Despite these promising attributes, the effects of ZLE and tricin on AD or astrocyte function have not yet been explored. To address this gap, the present study aimed to evaluate the regulatory effects of ZLE and tricin on astrocyte-related protein expression in C6 cells, which were subjected to conditions mimicking astrodegeneration characterized by reduced levels of GFAP and AQP4. Additionally, the study extended its analysis to an *in vivo* model using scopolamine-treated mice, a common approach in the development and testing of functional food ingredients, to assess the potential of ZLE and tricin in modulating neuroprotective mechanisms and astrocyte function.

## Materials and Methods

### Reagents

MTT (3-(4,5-dimethylthiazol-2-yl)2-,5-diphenyltetrazolium bromide), and insulin were purchased from Sigma-Aldrich (USA). Antibody against AQP4 (#59678), β-actin (#3700), GFAP (#80788), LRP1 (#64099), horseradish peroxidase-linked anti-mouse secondary antibody (#7076), horseradish peroxidase-linked anti-rabbit secondary antibody (#7074) were purchased from Cell Signaling Technology (USA). Antibody against BDNF (ab108319), matrix metalloproteinase (MMP)-2 (ab92536), MMP-9 (ab76003), and Aβ_1-42_ (Aβ_1-42_, ab120301) were purchased from Abcam Biotechnology (UK). IDE (sc-393887) was purchased from Santa Cruz Biotechnology (USA). Fetal bovine serum (FBS), Dulbecco’s modified Eagle’s medium (DMEM) were purchased from the American Type Culture Collection (USA).

### Cell Culture

C6 cells were purchased from American Type Culture Collection. Cells were cultured in DMEM supplemented with penicillin (120 units/ml), streptomycin (120 units/ml), and 10% FBS in a 5% CO_2_ atmosphere at 37°C.

### Animals

Balb/cJ mice (11 weeks old, male) were purchased from Hana Bio (Republic of Korea), and were used as the experimental animals after undergoing quarantine and acclimatization for a week at the Dongnam Medical Research Institute Animal Company (Animal Facility Registration Certificate: No. 412). During breeding, the lighting time was set to a 12-h cycle, and food and water were freely available. Group separation and treatment of experimental animals were designed and performed as presented in Table 1. This study was conducted by the Animal Experimental Ethics Committee of Dongnam Medical Research Institute (No. SEMI-21-003). Briefly, scopolamine (3 mg/kg, i.p.) was administered daily along with donepezil (0.5 mg/kg, positive control) for 7 days, after which the experimental animals were euthanized with CO_2_.

### Contents of Acetylcholine (ACh) and Acetylcholine Esterase (AChE)

The brain tissue was harvested and homogenized by adding 0.1 M PBS at a ratio of 1:10, respectively. After centrifugation at 10,000 ×*g* for 10 min, the resultant supernatant was separated, and the ACh content was measured using a mouse ACh ELISA kit (Cat No. #E4453-100, Biovision, USA). To measure the AChE activity, 5 μl of the brain tissue enzyme was dispensed in a 96-well plate, followed by addition of 150 μl 0.1 M Tris buffer (pH 8.0). Subsequently, 10 μl 0.01 M dithionitrobenzoic acid (DTNB) and 5 μl 0.1 M acetylthiocholine iodide were added to each well, followed by measurement of the absorbance at 405 nm. After 5 minutes, the absorbance was re-measured under the same conditions to confirm any changes in the absorbance.

### Hematoxylin and Eosin (H&E) Staining

The mice were sacrificed and brain tissues were harvested for further examination. Each tissue was fixed in formalin solution (4% paraformaldehyde in PBS). Then, the tissues were successively dehydrated in ethanol and xylene, followed by paraffin embedding. Each paraffin block contained a whole-brain sample, and was coronally sectioned to 5 μm thickness using a microtome (RM-2125 RT, Leica, Germany). The sliced sections were stained with H&E and mounted, followed by imaging using an optical microscope (Eclipse 80i, Nikon, Japan).

### Western Blot

C6 cells were seeded into a 6-well cell culture plate (1 × 10^5^ cells per well, cell passage; #75) for 24 h, and then treated with ZLE (50, 500 μg/ml) or tricin (1 μg/ml) with insulin (100 μg/ml) and aggregated Aβ_1-42_ (5 μM) for 48 h. C6 cells were lysed in a membrane protein extraction kit (Thermo Fisher Scientific, Inc., USA) or RIPA lysis buffer. In the *in vivo* model, the harvested brain tissue was ground in a tissue extraction solution (78510, Thermo Fisher Scientific) using a grinder, and subsequently centrifuged to obtain the proteins. Membrane protein or total protein concentrations were measured using a BCA protein assay (Thermo Fisher Scientific). Membrane proteins (100 μg) or total protein (50 μg for C6 cells; 20 μg for brain tissue) were separated by 12% sodium dodecyl sulfate-polyacrylamide gel electrophoresis and then transferred to PVDF membranes (Whatman GmbH, Germany). The transferred proteins were blocked with 5% skimmed milk in Tris-buffered saline containing 0.1% Tween-20 for 1 h and then incubated with AQP4, IDE, LRP1, BDNF, GFAP, MMP-2, MMP-9, β-actin for overnight at 4°C. After three washes in Tris-buffered saline containing 0.1% Tween-20, membranes were incubated with horseradish peroxidase-linked anti-mouse secondary antibody for 1 h. Proteins were detected by enhanced chemiluminescence and visualized using image software (UVP Vision Works^®^ LS Image Acquisition & Analysis Software, USA).

### Statistical Analysis

All experiments were repeated at least three times, and each experiment was performed in triplicate. Results are presented as means ± standard deviations (SD). A significant difference from the respective control for each experimental test condition was assessed using Student’s *t*-test for each paired experiment and *p* < 0.05 was considered to be significant.

## Results

### Effects of ZLE or Tricin on Astrocyte Proteins Expression in Aβ Plus High-Dose Insulin-Treated C6 Cells

**Fig. 1:**
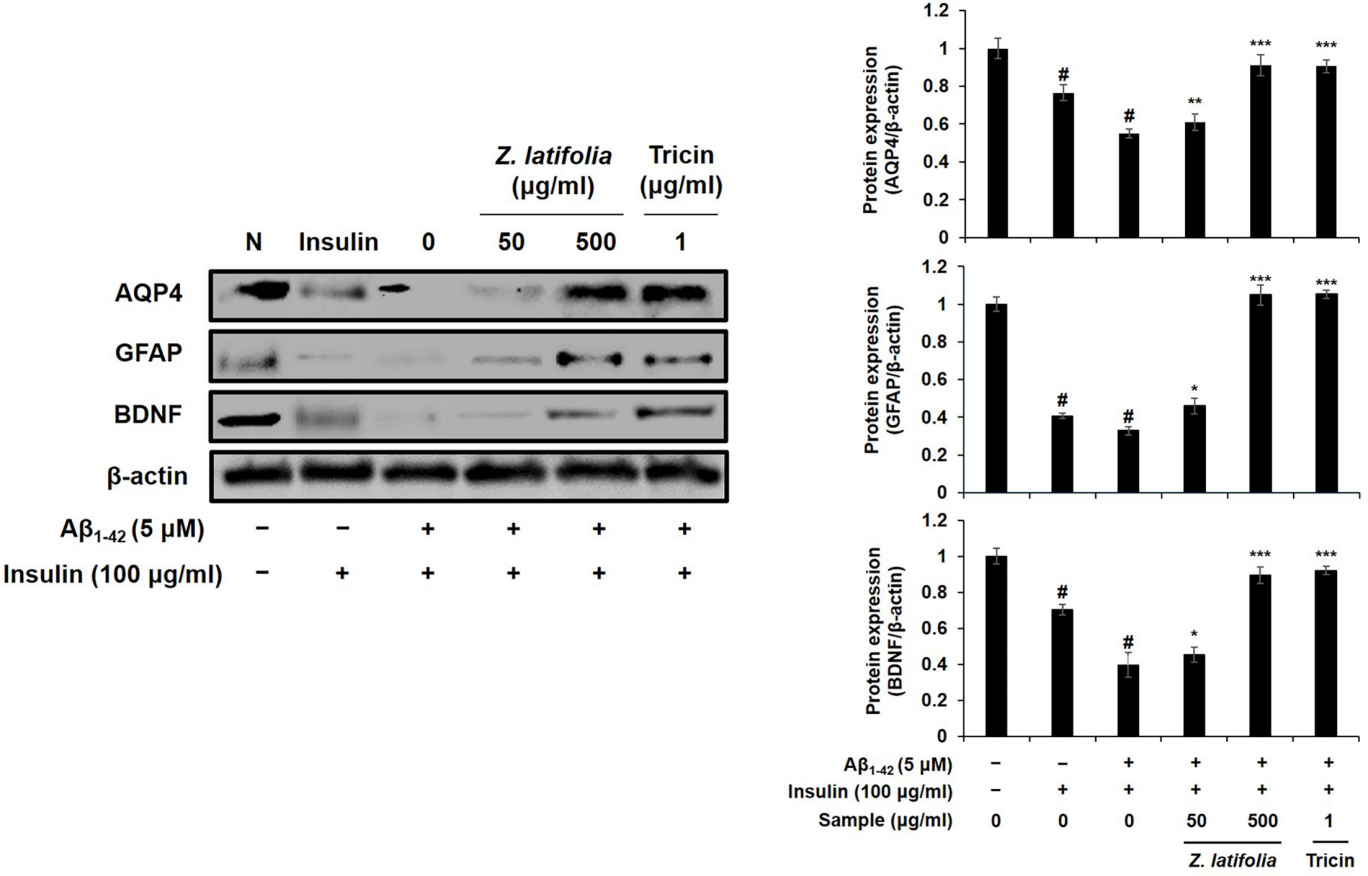
Effects of ZLE and tricin on AQP4, GFAP, and BDNF protein expression in C6 cells treated with highdose insulin and Aβ_1-42_. β-actin was used as the internal control for Western blot analysis. Results are presented as the means ± SD of percentages calculated with respect to control levels, of three independent experiments. ^#^*p* < 0.05, vs. untreated cell and *p < 0.05, *p < 0.005, ***p < 0.001 vs. insulin and Aβ-treated control.

First, to investigate the effect of ZLE or tricin on astrocyte dysfunction, the changes in the protein levels of AQP4, GFAP, and BDNF were investigated using Aβ plus high-dose insulin-treated C6 cells. Astrocytic condition of C6 cells for normal control were confirmed by the expression of GFAP and AQP4, together with the normal expression level of BDNF (Fig. 1, left panel, lane 1). Exposure of the cells to high-dose insulin (100 μg/ml) significantly suppressed the protein expressions of AQP4, GFAP, and BDNF in the C6 cells (Fig. 1, lane 2), and treatment with Aβ_1-42_ further inhibited these expressions (Fig. 1, lane 3). These suppressed expressions of AQP4, GFAP, and BDNF were reversed by the treatment of ZLE (500 μg/ml) about 1.65-, 3.19-, and 2.26-folds, respectively (Fig. 1, lane 5). Tricin (1 μg/ml) also up-regulated these protein expressions about 1.64-, 3.20-, 2.32-folds, respectively (Fig. 1, lane 6).

**Fig. 2:**
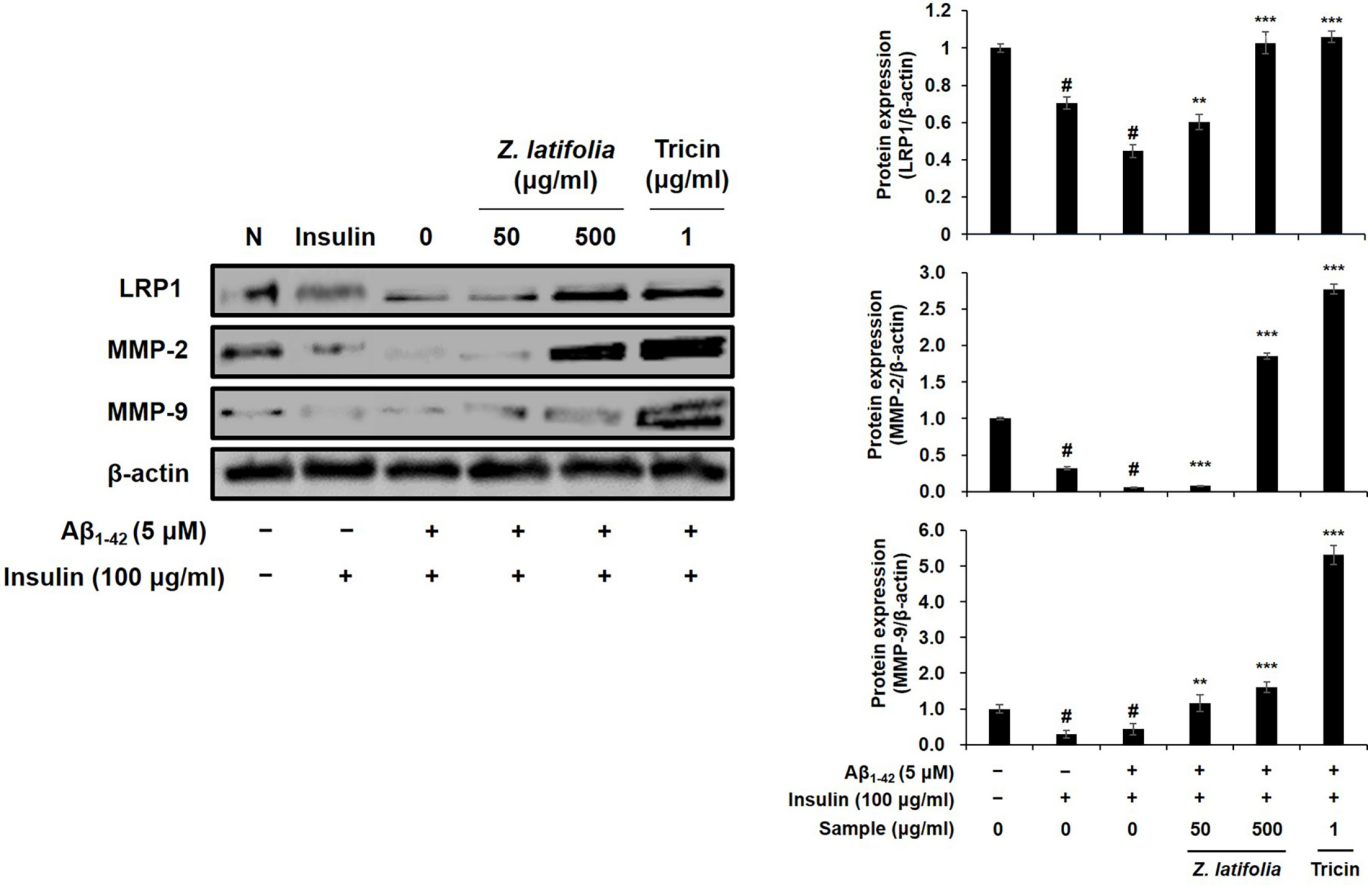
Effects of ZLE and tricin on LRP1, MMP-2, and MMP-9 protein expression in C6 cells treated with high-dose insulin and Aβ_1-42_. β-actin was used as the internal control for Western blot analysis. Results are presented as the means ± SD of percentages calculated with respect to control levels, of three independent experiments. ^#^*p* < 0.05, vs. untreated cell and *p < 0.005, ***p < 0.001 vs. insulin and Aβ-treated control.

We next examined the effects of the samples on the LRP1/MMPs-mediated Aβ clearance pathway in Aβ plus high-dose insulin-treated C6 cells. The LRP1 expression in astrocytes plays strategic roles in brain Aβ degradation [11], and LRP1 regulates expressions of the Aβ degradation enzymes MMP-2 and -9, eventually alleviating the accumulation of Aβ. Treatment the cells with Aβ plus high-dose insulin almost completely blocked the expressions of LRP1 and MMPs (Fig. 2, left panel, lane 3). The protein expressions of LRP1, MMP-2, and MMP-9 were significantly up-regulated about 2.30-, 32.07-, 3.74-folds, respectively, by ZLE (500 μg/ml, Fig. 2, lane 5), and 2.37-, 47.79-, and 12.37-folds, respectably, by tricin (1 μg/ml, Fig. 2, lane 6). These results suggest that ZLE and tricin could protect brain cells such as astrocytes against damage induced by high-dose insulin and/or Aβ, results in prevention of pathological aging of the brain as well as neurodegenerative disease, including AD.

### Effects of ZLE or Tricin on ACh and AChE in Brain Tissues of Scopolamine-Treated Mice

**Fig. 3:**
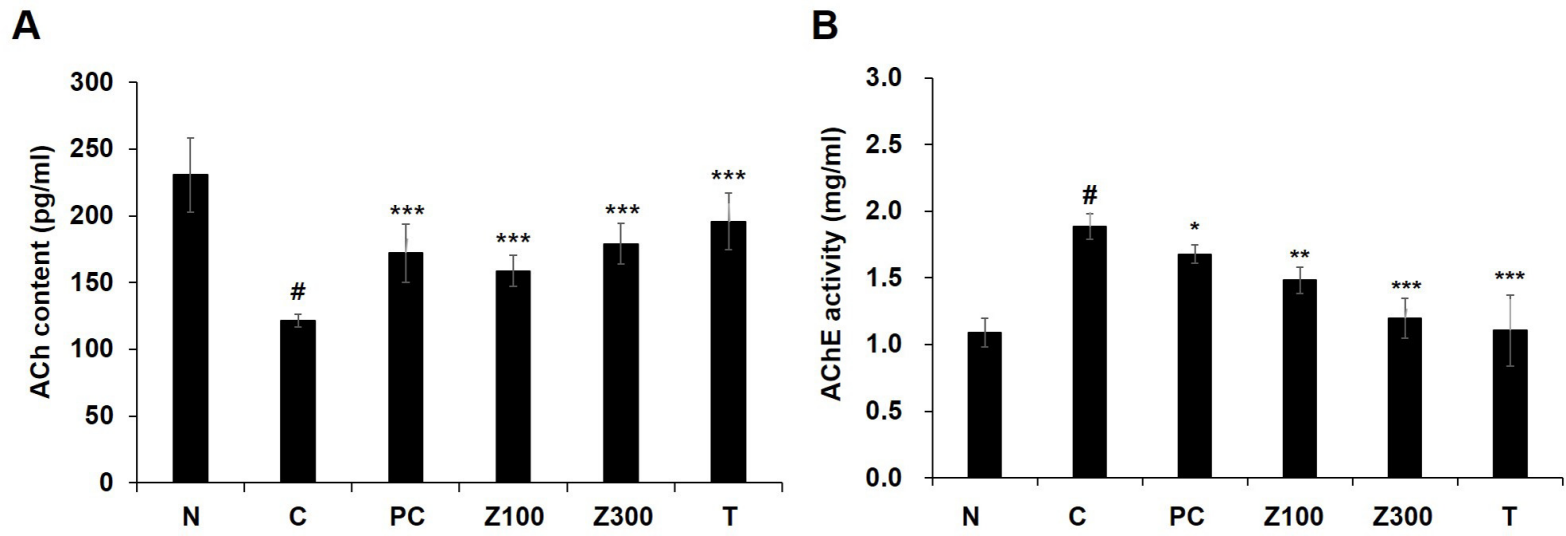
Effects of ZLE and tricin on ACh concentration and AChE activity in the hippocampus of scopolamine-treated mice. (**A**) ACh concentration ; (**B**) AChE activity. Results are presented as the means ± SD of percentages calculated with respect to control levels, of three independent experiments. ^#^*p* < 0.05, vs. normal group and *p < 0.05, *p < 0.005, ***p < 0.001 vs. scopolamine-treated group. Donepezil (5 mg/kg) was used as the positive control. N: normal group, C: scopolamine-treated control group, PC: donepezil-treated positive control group, Z100: ZLE 100 mg/kg treated group, Z300: ZLE 300 mg/kg treated group, T: tricin 0.3 mg/kg treated group.

To check that the administration of ZLE or tricin regulated acetylcholine (ACh) and acetylcholinesterase (AChE) in scopolamine-treated mice, ACh level and AChE activity were measured in mice brain tissues. As shown in Fig. 3A, the level of ACh in scopolamine-treated mice was significantly decreased (*p* < 0.001), and the administration of ZLE or tricin up-regulated significantly the ACh concentration (*p* < 0.001). In contrast, the activity of AChE in scopolamine-treated mice brain was increased about 1.73-fold compared to the normal group. Administration of ZLE (300 mg/kg) or tricin significantly returned to levels in the normal group (*p* < 0.001, Fig. 3B).

### Effect of ZLE or Tricin on Histopathological Changes of Scopolamine-Treated Mice

**Fig. 4:**
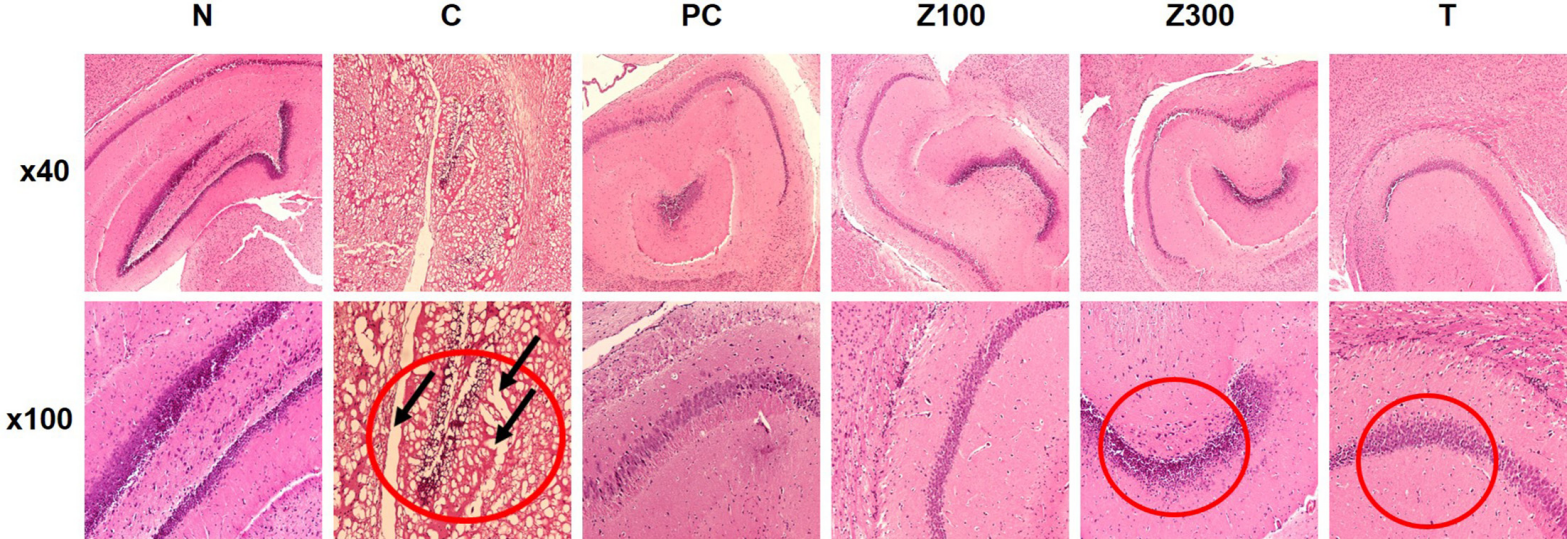
Histological examination by H&E staining in the hippocampus of scopolamine-treated mice administrated ZLE and tricin. Histological changes were observed at x40 (upper panels) and x100 (lower panels). Black arrows indicate brain tissue necrosis. Donepezil (5 mg/kg) was used as the positive control. N: normal group, C: scopolamine (3 mg/kg) treated control group, PC: scopolamine (3 mg/kg)+donepezil (5 mg/kg) treated positive control group, Z100: scopolamine (3 mg/kg)+ZLE (100 mg/kg) treated group, Z300: scopolamine (3 mg/kg)+ZLE (300 mg/kg) treated group, T: scopolamine (3 mg/kg)+tricin (0.3 mg/kg) treated group.

The results of the H&E staining for histopathological examination are shown in Fig. 4. The hippocampal neurons of the normal group exhibited well-arranged and a clear structure compared to the scopolamine-treated group. A clear pathological change was observed in the hippocampus of scopolamine-treated negative control group mice. The irregular or altered morphology was clearly prevented by the treatment with ZLE (100, 300 mg/kg) or tricin (0.3 mg/kg; corresponding of 300 mg/kg administration of ZLE group), suggesting the protective effect of ZLE- or tricin-administration against neuronal damage induced by scopolamine.

### Effect of ZLE or Tricin on Astrocyte Proteins Expression in Brain Tissues of Scopolamine-Treated Mice

**Fig. 5:**
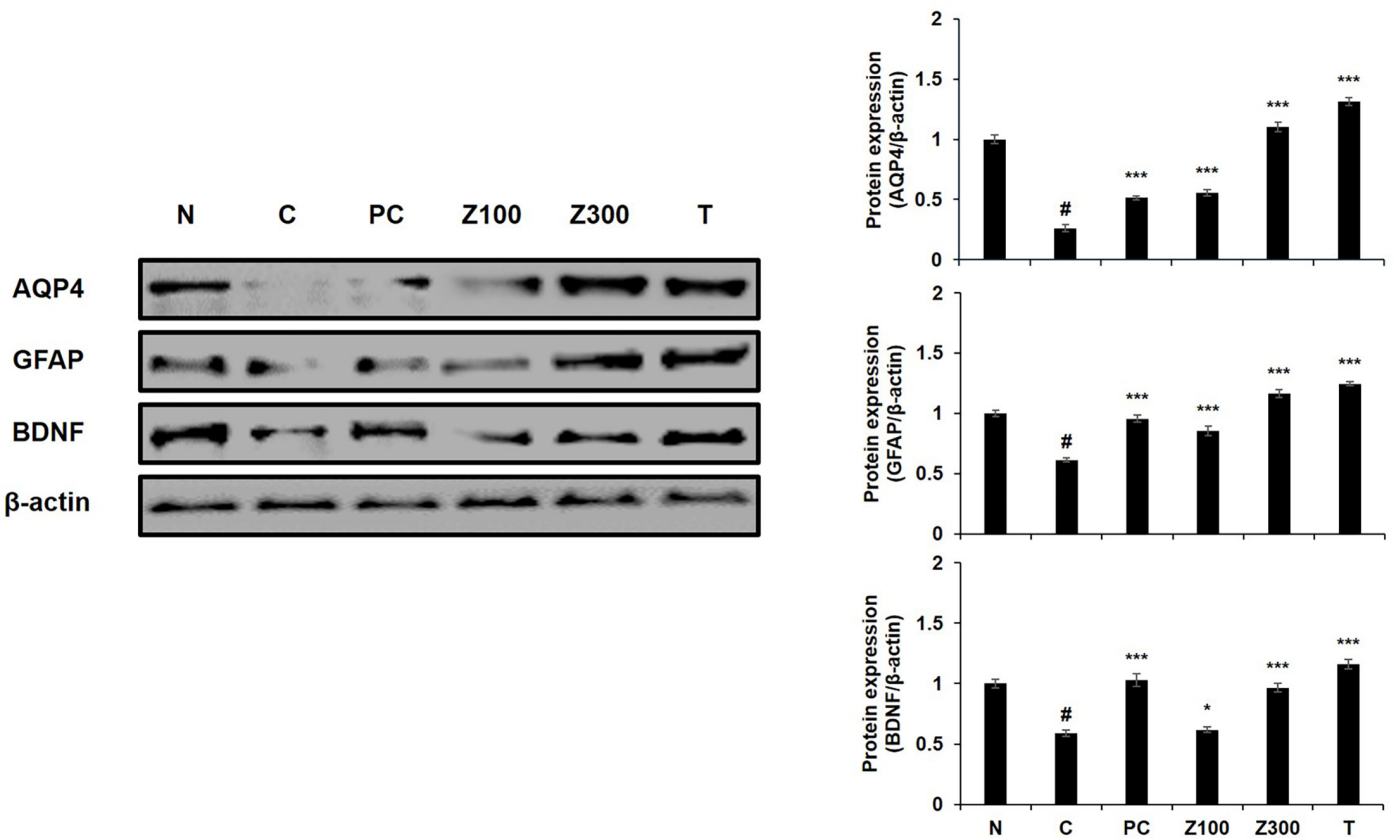
Effects of ZLE and tricin on AQP4, GFAP, and BDNF protein expression in scopolamine-treated mice. β-actin was used as the internal control for Western blot analysis. Results are presented as the means ± SD of percentages calculated with respect to control levels, of three independent experiments. ^#^*p* < 0.05, vs. normal group and *p < 0.05, ***p < 0.001 vs. scopolamine-treated group. Donepezil (5 mg/kg) was used as the positive control. N: normal group, C: scopolamine-treated control group, PC: donepezil-treated positive control group, Z100: ZLE 100 mg/kg treated group, Z300: ZLE 300 mg/kg treated group, T: tricin 0.3 mg/kg treated group.

The levels of AQP4, GFAP, and BDNF in hippocampus of scopolamine-treated mice were investigated by Western blot analysis. The expressions of these proteins were dramatically decreased in the scopolamine-treated control group (Fig. 5, left panel lane 2). However, the administration of ZLE at 300 mg/kg or tricin restored these proteins expression compared to the scopolamine-treated mice in the negative control group (*p* < 0.001, Fig. 5, lane 5 and 6). The administration of ZLE at 100 mg/kg also enhanced the expression of AQP4 and GFAP, while there was no significant effect on BDNF expression (Fig. 5, lane 4). Additionally, the donepezil-administrated positive control group was also displayed recovered protein expression of AQP4, GFAP, and BDNF (Fig. 5, lane 3).

**Fig. 6:**
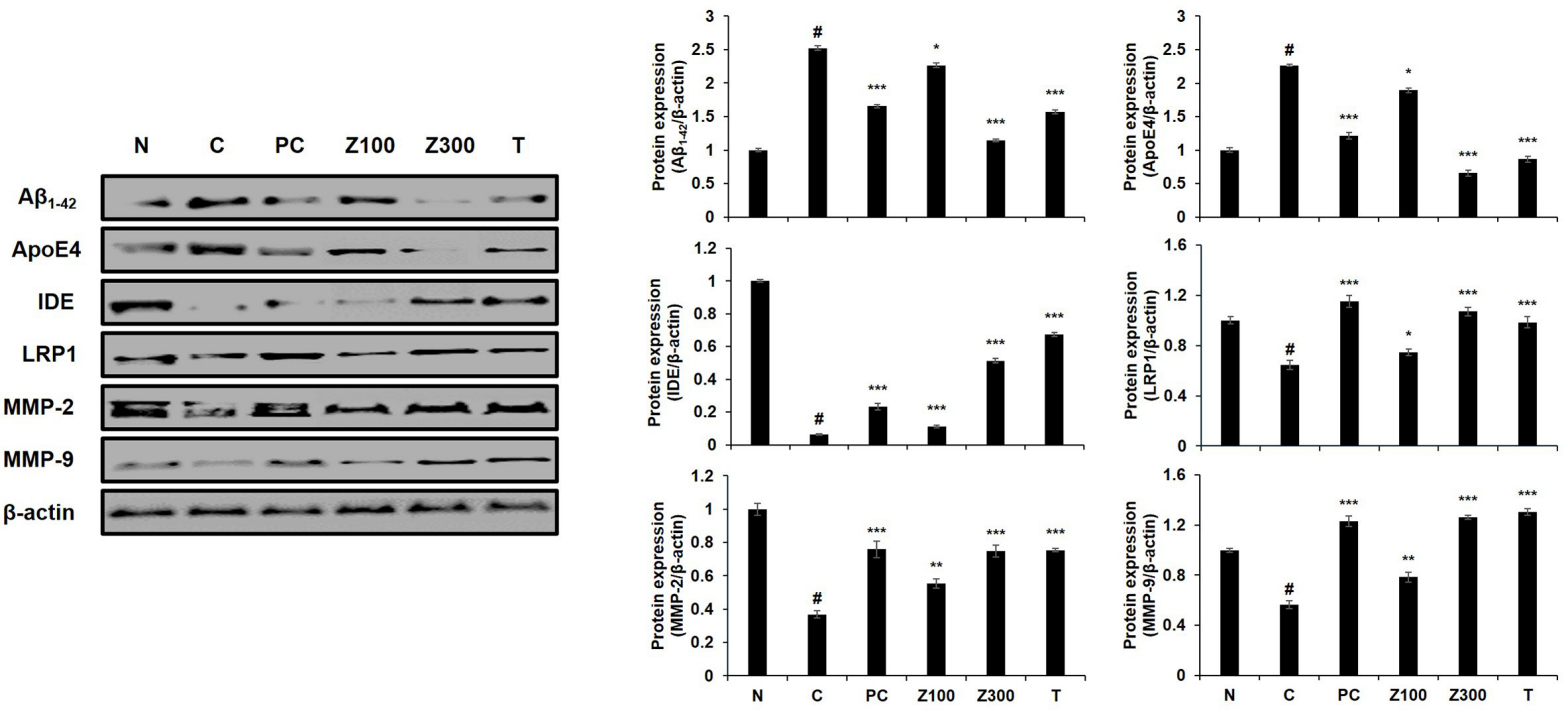
Effects of ZLE and tricin on Aβ_1-42_, ApoE4, IDE, LRP1, MMP-2, and MMP-9 protein expression in scopolamine-treated mice. β-actin was used as the internal control for Western blot analysis. Results are presented as the means ± SD of percentages calculated with respect to control levels, of three independent experiments. ^#^*p* < 0.05, vs. normal group and *p < 0.05, *p < 0.005, ***p < 0.001 vs. scopolamine-treated group. Donepezil (5 mg/kg) was used as the positive control. N: normal group, C: scopolamine-treated control group, PC: donepezil-treated positive control group, Z100: ZLE 100 mg/kg treated group, Z300: ZLE 300 mg/kg treated group, T: tricin 0.3 mg/kg treated group.

We next investigated the effects of ZLE or tricin on Aβ clearance pathway, such as LRP1 and Aβ degrading enzymes in scopolamine-treated mice. As shown in Fig. 6, the expression levels of Aβ uptake protein LRP1 and Aβ degradation enzymes (IDE, MMP-2, MMP-9) were suppressed in scopolamine-treated mice, but it was confirmed that these expressions were clearly increased in ZLE (300 mg/kg)- and tricin (0.3 mg/kg)-administrated mice as well as donepezil-administered control group compared with scopolamine-treated mice. On the other hand, the increased expression of ApoE4 protein induced by scopolamine treatment was reduced following sample administration, with a particularly significant reduction observed with Z300 and tricin treatment. These results suggest that in the scopolamine-induced amnesic condition, the generation of apoE4 fragments was promoted, potentially leading to amyloid-beta accumulation, which indicates impaired amyloid clearance.

## Discussion

This study aimed to assess the effects of ZLE and its active compound, tricin, on astrocyte dysregulation induced by high-dose insulin combined with Aβ_1-42_ in C6 astroglial cells. The C6 cell line, which shares characteristics with astrocytes, is commonly utilized *in vitro* to model conditions associated with Alzheimer’s disease (AD), making it a valuable tool for studying glial cell behavior and responses under experimental aging conditions [25, 38, 39]. Aβ peptides have been extensively used in both *in vitro* and *in vivo* studies to create AD-like environments, leading to outcomes such as neuronal cell death, reduced expression of astrocyte marker proteins, and inflammation, depending on the concentration and type of Aβ peptide used [3, 40]. Insulin, recognized as an important neurotrophic factor, plays a key role in brain function by influencing synaptic plasticity, learning, memory, and neurotransmitter regulation across various receptor sites [41]. However, insulin also has the potential to increase neuronal susceptibility to excitotoxicity. For example, in rat neuron cultures, insulin has been shown to amplify the cytotoxic effects of excitatory amino acids like glutamate, indicating potential toxicity at elevated concentrations [28, 29]. Moreover, studies in animal models have reported cognitive impairments linked to high-dose insulin exposure [42], which is consistent with the astrocyte dysfunction observed in C6 cells treated with high insulin levels. IDE plays a critical role in metabolizing insulin after it is internalized by cells, preventing its excessive buildup. Elevated insulin levels can compete with Aβ for degradation by IDE, thereby impairing the clearance of Aβ and contributing to the accumulation associated with AD pathology [31].

AQP4 plays a pivotal role in the degradation of Aβ and the uptake of glutamate in astrocytes, with its deficiency being strongly associated with cognitive impairments. Research has demonstrated that mice lacking the AQP4 gene exhibit increased deposition of Aβ plaques in the brain, accompanied by memory deficits [3, 4]. In our study, we observed that treatment with ZLE or its major compound, tricin, exerted a protective effect against astroglial dysfunction in C6 cells exposed to high-dose insulin and Aβ_1-42_. Specifically, we found that the upregulation of astroglial markers GFAP and AQP4 following treatment with ZLE or tricin may help mitigate deficits in the Aβ clearance pathway, as evidenced by the enhanced expression of LRP1 and MMPs, which are critical in the pathogenesis of AD. Tricin, a flavonoid present in the Gramineae family, was identified as the primary active compound in ZLE responsible for its protective effects, including the prevention of allergic responses. Prior research has identified five derivatives of tricin (4',5,7-trihydroxy-3',5'-dimethoxyflavone) in the methanol extract derived from the aerial parts of *Z. latifolia*, further highlighting its pharmacological significance [43].

In our previous research, we observed that the expression levels of astrocytic markers GFAP and AQP4 increased with successive cell passages. Consequently, for optimal protein expression, we selected cells at passage numbers 75 to 85. To simulate astrodegenerative conditions characterized by reduced GFAP and AQP4 expression, as reported in earlier studies [27], we treated the astroglial cells with a combination of high-dose insulin and Aβ_1-42_. The downregulation of GFAP and AQP4 in astrocytes may indicate dysfunction, and our findings suggest that ZLE and tricin have the potential to counteract AD progression by preserving astrocyte function, particularly within the Aβ clearance system. Our data demonstrated that ZLE and tricin effectively protected astrocytes from deregulation induced by high-dose insulin and Aβ_1-42_, leading to the upregulation of key proteins essential for astrocyte function (Fig. 1) and Aβ degradation (Fig. 2). Additionally, existing studies have established that astrocytic low-density lipoprotein receptor-related protein 1 (LRP1) is crucial for Aβ clearance in the brain [11, 12]. Knockdown of LRP1 in primary astrocytes has been shown to reduce cellular Aβ uptake and degradation, while also downregulating several major Aβ-degrading enzymes, including MMP-2, MMP-9, and IDE, further emphasizing the importance of LRP1 in maintaining Aβ homeostasis [44].

Additionally, we investigated the impact of ZLE and tricin on the expression of astrocyte marker proteins and the Aβ-degrading pathway in a scopolamine-induced mouse model. Scopolamine, a muscarinic acetylcholine receptor antagonist, disrupts cholinergic neurotransmission, leading to cognitive and memory impairments in animal models. These impairments mimic the memory dysfunction observed in Alzheimer's disease patients, making scopolamine a widely used agent for studying neurodegenerative processes and potential therapeutic interventions [45]. Scopolamine-induced elevation of AChE activity is linked to a reduction in ACh levels, a process that can result in memory deficits and is implicated in the development of AD pathology [46]. In this study, the administration of *Zizania latifolia* extract (ZLE) and tricin effectively mitigated scopolamine-induced alterations in acetylcholine (ACh) levels and acetylcholinesterase (AChE) activity (Fig. 3), as well as reducing brain tissue damage (Fig. 4). Histopathological analysis using H&E staining in the normal control group revealed no significant pathological lesions. In contrast, the scopolamine-treated control group exhibited abnormal morphological changes in the hippocampus, which were prevented by pretreatment with ZLE (100, 300 mg/kg) and tricin (0.3 mg/kg). Scopolamine-induced short-term memory loss in animal models, often associated with Alzheimer's disease, correlates with changes in the expression of proteins involved in Aβ generation and degradation. Our study found that exposure to scopolamine resulted in decreased expression of AQP4, GFAP, and BDNF, which were subsequently restored by the administration of ZLE and tricin (Fig. 5). These findings are consistent with previous research that reported a reduction in BDNF and GFAP expression in scopolamine-induced amnesia models, both in mice and in C6 cell cultures [47]. Additionally, the administration of ZLE at 300 mg/kg and tricin at 0.3 mg/kg effectively prevented the significant reduction in proteins associated with Aβ uptake (ApoE4, LRP1) and Aβ degradation (IDE, MMP-2, MMP-9) (Fig. 6). These results indicate that ZLE and tricin may play a critical role in regulating astrocyte function, both *in vitro* under astrodegenerative conditions and *in vivo*. Collectively, our findings suggest that ZLE and tricin have the potential to upregulate AQP4 expression, which could lead to enhanced Aβ clearance via AQP4-mediated degradation pathways. This mechanism may be particularly important in preventing astrodegenerative diseases, including the early stages of AD.

In conclusion, this study demonstrated the protective effects of ZLE and tricin on astrocyte function within an *in vitro* model that simulates early-stage AD, as evidenced by changes in marker expression corresponding to various stages of AD progression. However, to advance the development of ZLE extract containing tricin as a potential preventive agent for AD, and to establish AQP4 as a target under *in vitro* AD-like conditions, further investigation is required. Specifically, it is essential to validate the relationship between astrocyte marker protein expression and variables such as age and Aβ concentration using an Aβ-treated AD mouse model.

## Figures and Tables

**Table 1:** Experimental design.

Group	Mice	Treatment
CON	7	Saline (i.p) + saline (p.o)
SCO	7	Scopolamine 3 mg/kg (i.p) + saline (p.o)
DON	7	Scopolamine 3 mg/kg (i.p) + donepezil 5 mg/kg (p.o)
ZLE100	7	Scopolamine 3 mg/kg (i.p) + ZLE^1)^ 100 mg/kg (p.o)
ZLE300	7	Scopolamine 3 mg/kg (i.p) + ZLE^1)^ 300 mg/kg (p.o)
Tricin	7	Scopolamine 3 mg/kg (i.p) + tricin 0.3 mg/kg (p.o)

^1)^ZLE, *Zizania latifolia* extract
